# In vivo evaluation of hot water extract of *Acorus gramineus* root against benign prostatic hyperplasia

**DOI:** 10.1186/s12906-017-1887-9

**Published:** 2017-08-22

**Authors:** Joo-Myung Moon, Hae-Mi Sung, Hyun-Jung Jung, Jae-Won Seo, Ji-Hyang Wee

**Affiliations:** 1 #913, Technology advancement dong, Gyeonggi Technopark 705 Haean-ro, Sangnok-gu, Ansan-si, Gyeonggi-do 15588 Republic of Korea; 2 Food Research Center, Jeonnam Bio-industry Foundation, 30-5 Dongsunonggongdanji-gil, Naju-si, Jeollanam-do 58275 Republic of Korea

**Keywords:** *Acorus gramineus*, Benign prostatic hyperplasia, 5-α reductase, Anti-oxidant activation androgen receptor

## Abstract

**Background:**

*Acorus gramineus* has been reported to exhibit various pharmacological effects including inhibition of cholesterol synthesis, enhancement of lipid metabolism, prevention of dementia and inhibition of mast cell growth. According to the Chinese compendium of materia media, it has been reported that *Acorus spp.* is effective for sedation, dementia prevention as well as diuretic effect. In addition, it showed more than equivalent activity compared to furosoemide, a drug known to be effective in diuretic action in animal model study. However, their effectiveness against benign prostatic hyperplasia (BPH) of *Acorus gramineus* has not been reported. This study was designed to evaluate the effect of *Acorus gramineus* root hot water extract (AG) against BPH in vivo.

**Methods:**

Male rats, 10 weeks of age and weighing 405 g ± 10 g, were used for this study. Biomarkers were evaluated including prostate weight, prostate weight ratio, hormonal changes, 5-α reductase type II androgen receptor (AR) of the prostate gland and anti-oxidant activation factors related to BPH. These biomarkers were measured in vivo test.

**Results:**

AG showed significant effect at the 250 and 500 mg/kg/day in rats. Groups treated with AG displayed significantly lower levels of prostate gland weight (0.79 g) compared to the BPH induced group (1.19 g). Also, dihydrotestosterone (DHT) level was decreased from 61.8 to 100% and androgen receptor expression level was decreased from 111 to 658%. Any hematological toxicity of alanine aminotransferase (ALT) and aspartate aminotransferase (AST) level wasn’t observed.

**Conclusion:**

This study indicated that AG was effective for reducing BPH symptoms.

**Trial registration:**

Not applicable.

## Background

At andropause, the level of the male hormones such as testosterone decrease. In contrast, level of the female hormones like estrogen increase. Various symptoms occur including decreased bone density, muscle mass, concentration and sexual desire. Additionally, depression, headache, insomnia, fatigue, an increase in abdominal fat and a decrease in general physical strength occur [1–3].

Male hormone deficiency symptoms are called the Andropause syndrome which is described by six characteristic complex symptoms such as the decrease of sexual vigor, emotional changes, the increase of body fat, the decrease of body hair, osteoporosis and abdominal obesity [1–3].

Males enter andropause in their forties and experience the above mentioned symptoms in addition to benign prostatic hyperplasia, the gradational enlargement of the prostate gland [4]. An enlarged prostate gland suppresses the urethra where it enters the prostate and urine excretion is interrupted, causing inflammation.

BPH occurs more frequently than hypertension and diabetes. In fact, 40% of men in their forties, 50% of men in their fifties, 60% of men in their sixties and 70% of men in their seventies experience histological BPH. Of these, 25% experience clinical symptoms of BPH [5]. Pharmaceutical treatments of BPH have been classified as follows: α-1 inhibitors as improve urination; 5-α reductase inhibitors that reduce the prostate size. The representative drug, finasteride is 5-α reductase inhibitor used for BPH but it is associated with a variety of side effects [6].

*Acorus gramineus* is perennial plant that has green leaves during all four seasons. It is found in Korea, China, Japan and India [7]. The root and stem contain 0.5 ~ 0.8% essential oil and its main components are γ-aminobutyric acid (GABA), asarone, palmitic acid, phenol, calamenol, palmitin [8, 9].

Asarone has various pharmacological effects. α-asarone is effective for inhibiting cholesterol synthesis and enhancing lipid metabolism [10]. β-asarone is effective for preventing dementia and is used as a pharmaceutical ingredient. β-asarone was reported to inhibit cell growth and cause cell contraction when injected into mast [11, 12]. Some countries have registered *Acorus gramineus* as a toxic material because of β-asasone. However, Korean Food Standard Codex permits the water extract for use in food [13]. We removed most of the β-asasone in *Acorus gramineus* by hot water extraction*.*

This study was designed to evaluate the effectiveness of *Acorus gramineus* root hot water extract (AG) for the treatment of BPH in vivo.

## Methods

### Material preparation and analytic method

#### Instruments

Extraction concentrator was purchased from Seugyung Eng. (Ansan, Korea). Agilent Infinity 1260 was used for qualitative and quantitative analysis of *Acorus gramineus* extract.

#### Materials and reagents

Methanol and acetonitrile were purchased from J.T. & Baker (Pennsylvania, USA). Hydrochloride was purchased from JUNSEI (Tokyo, Japan). Phosphoric acid and sodium phosphate dibasic anhydrous (Na_2_HPO_4_) were purchased from SAMCHUN (Sung-nam, Korea). Borate buffer (3-mercaptopropionic acid in borate buffer) and OPA (Ortho-Phthal aldehyde) was purchased from Agilent (Santa Clara, (California), USA). GABA standard material was purchased from Sigma-Aldrich (St. Louis, (Missouri), USA). Finasteride was purchased from Tokyo Chemical Industry Co. Ltd. (Tokyo, Japan).

#### AG extraction method

*Acorus gramineus* root was purchased from Umji, cultivated at Jeju Island in Korea and harvested in September 2014. *Acorus gramineus* was confirmed by BTC R&D center in accordance with the confirmation test method of The Korean Herbal Pharmacopoeia. A voucher specimen was deposited at the Herbarium of the ChonBuk National University, Republic Of Korea. AG was produced as follows: 1 kg of dried *Acorus gramineus* root and 20 kg of water were placed into the extraction concentrator and extracted for 6 h at 90 °C. Then extract was filtered by 200 mesh net. The filterate was then concentrated using an evaporator at 65 °C and dried using a vacuum dryer (Ilshin Corp., Korea). A total of 215 g of extract powder was obtained. Based on the HPLC analysis, GABA was selected as the indicator material in *Acorus gramineus*

### Analytic method

#### Sample solution preparation

GABA consists of single bond so it cannot absorb UV/Vis light. Because of this, it is necessary to derivatize a compound that has absorption ability and HCl was used for derivatization. Distilled water 1 L included 8.8 mL of 35% HCl were combined in 1 L volumetric flask and 1.2 g of AG was mixed for 10 min.

#### Standard solution preparation

GABA standard 250 mg was added to a 250 mL flask and dissolved by solution that made to sample solution. Then, the mixture was shaken for 5 min with an ultrasonic shaker. The resulting solution was diluted to the standard solution concentrations: 50, 80, 100, 150, 200 μg/mL.

#### Derivatization and HPLC-DAD analyzing conditions

The hydrolyzed sample was automatically derivatized with OPA (Ortho-Phthal aldehyde) in accordance with the agilent autosampler program. An amount of 36.5 μL sample was injected on a Zorbax Eclipse AAA column, 4.6 × 150 mm, particle size 3.5 μm (Agilent, USA), at 40 °C. The mobile phase (A) 40 mM Na_2_HPO_4_ in 0.1% H_3_PO_4_ in distilled water and (B) acetonitrile was applied as follows: 0.0–0.9 min, 15% B; 1.9–10.0 min, 15–57% B; 10.0–10.5 min, 57–80% B; 10.5–13.0 min, 80% B and re-equilibration of the column over 15 min. The flow rate was 1.5 mL min^−1^ and diode array detector (DAD) was used for acquiring chromatograms at 338 nm.

### Experimental animal treatment

Experimental rats (age 10 weeks, weight 405 ± 10 g) were purchased from Orient-Bio Ltd. (Sung-nam, Korea). Animals were housed at 22 ± 2 °C, with 55 ± 5% humidity and a 12 h dark/light cycle. They were provided access to food and water ad libitum. The institutional Animal Care and Use Committee National University (Kwangju, Korea) approved the protocol for the animal study and the animals were cared for in accordance with the “Guidelines for Animal Experiment” established by the university.

### BPH induction and treatment

Rats were randomly divided into a control group (*n* = 7) and a BPH induced group (*n* = 35). To induce BPH, 3 mg/kg of testosterone propionate (TP) was injected daily for 4 weeks [14].

The BPH induced group was further divided into a control group, an AG medicated group and finasteride-treated group. These groups were fed AG (100, 250, or 500 mg/kg daily) and finasteride (10 mg/kg daily) respectively by oral ingestion over 4 weeks.

### Castration

After maintained 1 week, animals were castrated to remove any internal testosterone influence. The rats were anesthetized with CO_2_ and ether, both sides of the scrotum were incised to expose the testicles and the epididymis and the spermatic cord, vascular tracts and incised part were sutured.

Castration was executed referring to the OECD Hershberger assay method provided by the Institute of National Toxicity Laboratory. One week after castration, the rats were used for experiments [15].

### Measurement of body weight and feeding efficiency

Initial body weight was recorded 1 week after castration. During the 8 weeks experimental period, body weight was measured every 3 days and the TP injection quantity and feeding quantity were recorded.

Prior to dissection, the rats were fasted for 12 h and a final body weight was recorded. Body weight variation in the experimental animals was described as weight gain. Feeding efficiency was calculated using the food efficiency ratio (FER) based on the feeding quantity of the experimental animals [16].

### Organ weight and storage

Rats were anesthetized with CO_2_ and the abdomen was cut open to expose the organs. The organs were weighed then stored at -80 °C after being washed once with 1× PBS solution [17].

### Analysis of plasma alanine aminotransferase (ALT) and aspartate aminotransferase (AST)

Experimental animal serum was separated from blood after centrifugation at 890×*g*. ALT and AST were measured at 505 nm wave length using spectrophotometer (MQS200R; BioTek Instruments, Inc., Winooski, VT, USA). The results were converted to Karmen units and compared to each other [18].

### Measurement of testosterone and DHT in blood

Testosterone content was measured with an assay kit (Enzo Life Sciences, Farmingdale, NY, USA) using the following method. Serum (100 μL) was mixed with testosterone enzyme immunoassay (EIA) antibody (50 μL) and cultured for 1 h. Thereafter, 50 μL of conjugate was added and the solution was cultured for 1 h. After washing with washing solution, 200 μL of pNpp substrate solution was added and allowed to for 1 h. 50 μL of stop solution, solution of trisodium phosphate in water, was then added to stop the reaction and the absorbance was measured at 405 nm wave length. Testosterone content was calculated using a testosterone standard curve.

DHT content was measured using an assay kit (Biovendor, Brno, Czech Republic). Serum (50 μL) was mixed with conjugate (100 μL) and left for 1 h at 25 °C. The mixture was washed three times with washing buffer, then 150 μL of 3,3’,5,5’-Tetramethylbenzidine (TMB) substrate was added and the mixture was allowed to react for 15 min. Thereafter, 50 μL of stop solution was added to stop the reaction and the absorbance was measured at 405 nm wavelength. DHT content was calculated using a DHT standard curve [19].

### Measurement of gene expression rate in the prostate

Gene expression in the prostatic gland was measured using a real-time polymerase chain reaction (RT-PCR) method. After washing the exposed organ with 1× PBS, 0.5 mL of TRIzol reagent (Life technologies, Carlsbad, CA) was used to extract the RNA. cDNA was synthesized as follows: 1 μg of extracted RNA, 2 μL of gDNA buffer, 1 μL of reverse transcriptase, 4 μL of RT buffer and 1 μL of RT primer was mixed and allowed to react at 42 °C for 50 min, then at 70 °C for 15 min.

Relative mRNA levels of prostate genes, including 5-α reductase type I, 5-α reductase type II, AR and the inflammatory genes, IL-1β (Interleukin-1β), IL-6, COX-2 (Cyclooxygenase-2) and iNOS (inducible nitric oxide synthase), were measured using RT-PCR with a Rotor-Gene SYBR Green PCR kit (Qiagen, Hilden, Germany). Finasteride was used as a positive control [20]. Quantitative PCR amplification was performed in triplicate using the QuantiTect Promer assay (QT00070518 for SRD5A2, QT00073451 for AR and QT01680476 for ACTB; Qiagen).

IL-1β, IL-6, COX-2 and iNOS PCR primers were as follows: IL-1β, Forward primer: 5′-TTCGACACATGGGATAACGA-3′, Reverse primer: 5′-TCTTTCAACACGCAGGACAG-3′; IL-6, Forward primer: 5′-TACCCCCAGGAGAAGATTCC-3′, Reverse primer: 5′-TTTTCTGCCAGTGCCTCTTT-3′; COX-2, Forward primer: 5′-TGCTGTGGAGCTGTATCCTG-3′, Reverse primer: 5′-CGGGAAGAACTTGCATTGAT-3′ and iNOS, Forward primer: 5′-CTCACTGGGACTGCACAGAA-3′, Reverse primer: 5′-GCTTGTCTCTGGGTCCTCTG-3′.

### Measurement of protein expression rate in the prostate

Protein expression rate in the prostate gland was measured by sodium dodecyl sulfate-polyacrylamide gel electrophoresis (SDS-PAGE). After washing the exposed prostate gland with 1× PBS (Phosphate buffered saline), KCl lysis buffer was added, allowed to react at 4 °C for 1 h and centrifuged at 16600×*g* for 20 min at 4 °C. The supernatant was collected and the protein concentration was measured using a BCA (Bicinchoninate) protein quantification method (Amersham, Waltham, MA, USA).

Thirty micrograms of total protein extract was loaded on 4–10% polyacrylamide gel (Life Technologies, Seoul, Korea) for SDS-PAGE. Separated proteins were transferred to PVDF (Polyvinylidene difluoride) membranes (Life Technologies). Blocking was performed using TBS-T (Tris-buffered saline) buffer (0.1% Tween 20, 5% bovine serum albumin) on a shaker for 1 h at 4 °C, then the membrane was washed with TBS-T buffer. COX-2, phosphorylated NF-κB (Nuclear factor kappa B), NF-κB, 5-α reductase type II and β-actin antibody (Cell Signaling Technology, Beverly, MA, USA) were diluted 1:1000 and attached for 16 h at 4 °C. After washing the membrane, anti-Rabbit IgG secondary antibody (Cell Signaling Technology) was attached and the membrane was re-washed. Finally, Amersham ECL (Enhanced chemiluminescence) prime western blotting detection reagent was used to treat the membrane and results were analyzed using an image reader (Supernova 1800, Lugen Sci So., LTD. Bucheon, Korea) [21].

### Hematoxylin & eosin (H&E) staining

The prostate glands were sliced to an approximately 10 μm thickness, attached to slides and left for 5 min. The slides were then washed with flowing water for 5 min, prepared with 94% ethyl alcohol for 1 min and washed with flowing water for 30 s. The slices were stained with hematoxylin for 1 min, washed in flowing water for 30 s, then stained with eosin for 30 s. The slices were again washed with 94% ethyl alcohol two times for 30 s, treated with absolute alcohol for 30 s, washed with xylene for 30 s and washed with flowing water for 30 s [15].

### Statistical analysis

Results are expressed as mean ± standard error of the mean (SEM). Data were analyzed using one-way analysis of variance (ANOVA). Differences in each group were considered significant at *p* < 0.05 by Duncan’s multiple range test.

## Results

### Change in body weight and organ weight

#### Body weight and feeding efficiency

In case of BPH induced group, it was reduced to a significant reduction in weight about 47.7 g compared to control group. After the end of the experiment, measured weight was 451.6 ± 11.3, 459.0 ± 11.0, 444.9 ± 5.1 and 455.5 ± 17.4 g in the AG 100, 250 and 500 mg/kg/day and finasteride treated groups, respectively. Table 1 shows body weight and feeding efficiency ratio.

**Table 1:** Initial and final body weight, change of body weight and feeding efficiency

Group	No. of Rats	Initial BW (g)	Final BW (g)	BW Gain (g)	FER (%)	Survival Rate (%)
Control	7	404.6 ± 2.4^a^	534.4 ± 12.3^b^	111.8 ± 10.2^b^	1.00 ± 0.09^b^	100
BPH	7	406.5 ± 9.2^a^	470.6 ± 18.4^a^	64.1 ± 11.6^a^	0.41 ± 0.07^a^	100
BPH + AG 100 mg	7	405.0 ± 8.2^a^	451.6 ± 11.3^a^	46.6 ± 7.2^a^	0.30 ± 0.04^a^	100
BPH + AG 250 mg	7	406.2 ± 8.8^a^	459.0 ± 11.0^a^	52.9 ± 4.5^a^	0.35 ± 0.03^a^	100
BPH + AG 500 mg	7	403.5 ± 4.3^a^	444.9 ± 5.1^a^	41.4 ± 5.3^a^	0.26 ± 0.03^a^	100
Finasteride	7	405.1 ± 9.3^a^	455.5 ± 17.4^a^	50.5 ± 12.5^a^	0.33 ± 0.05^a^	100

Data express the mean ± S.E. The letters in the columns indicate statistical differences by Duncan’s multiple range test (*p* < 0.05). Control: Normal rat supplied with tap water, BPH: Castrated rat supplied with tap water, AG 100: Castrated rat supplied with AG 100 mg/kg of BW daily, AG 250: Castrated rat supplied with AG 250 mg/kg of BW daily, AG 500: Castrated rat supplied with AG 500 mg/kg of BW daily. Data are represented as mean±SEM. a, P<0.05 vs. control,  b, P<0.01 vs. control, c, P<0.001 vs. control

### Organ weight and weight ratio of the prostate gland

The weights of the liver, kidney and spleen were not significantly different among the control, BPH, finasteride-treated and AG-treated groups.

Prostate gland weight and weight ratio in the BPH induced group were significantly higher than those of the control group. However, prostate gland weight and weight ratio in the 250 and 500 mg/kg/day of AG and finasteride-treated group were significantly lower than those of the BPH induced group (Table 2).

**Table 2:** Organ weight and prostate weight ratio

Group	Liver (g)	Kidney (g)	Spleen (g)	Prostate (g)	Prostate ratio (mg/100 g of BW)
Control	11.48 ± 0.44^a^	3.81 ± 0.06^a^	0.65 ± 0.02^a^	0.59 ± 0.03^d^	0.13 ± 0.01^d^
BPH	11.39 ± 0.47^a^	3.75 ± 0.15^a^	0.64 ± 0.03^a^	1.19 ± 0.11^a^	0.26 ± 0.03^a^
BPH + AG 100 mg	11.66 ± 0.86^a^	3.50 ± 0.11^a^	0.62 ± 0.02^a^	0.99 ± 0.08^ab^	0.22 ± 0.03^ab^
BPH + AG 250 mg	12.39 ± 0.30 ^a^	3.70 ± 0.06^a^	0.69 ± 0.03^a^	0.87 ± 0.09^b^	0.19 ± 0.02^b^
BPH + AG 500 mg	11.19 ± 0.25^a^	3.50 ± 0.06^a^	0.69 ± 0.03^a^	0.79 ± 0.04^c^	0.18 ± 0.03^c^
Fisnasteride	11.85 ± 0.45^a^	3.57 ± 0.07^a^	0.70 ± 0.06^a^	0.82 ± 0.06^c^	0.18 ± 0.03^c^

Data express the mean ± S.E. The letters in the columns indicate statistical differences by Duncan’s multiple range test (*p* < 0.05). Control: Normal rat supplied with tap water, BPH: Castrated rat supplied with tap water, AG 100: Castrated rat supplied with AG 100 mg/kg of BW daily, AG 250: Castrated rat supplied with AG 250 mg/kg of BW daily, AG 500: Castrated rat supplied with AG 500 mg/kg of BW daily. Data are represented as mean±SEM. a, P<0.05 vs. control, b, P<0.01 vs. control, c, P<0.001 vs. control

### Toxicity and BPH hormones in blood

#### Toxicity estimation in the blood (ALT, AST)

ALT and AST values were not significantly different among the BPH, finasteride and AG-treated groups. Therefore, the results indicated that our samples were not toxic. Table 3 shows results of the toxicity evaluation.

**Table 3:** Blood toxicity estimation in the blood

	Control	BPH	BPH + AG 100 mg	BPH + AG 250 mg	BPH + AG 500 mg	Finasteride
AST	166.8 ± 6.1^a^	171.6 ± 5.4^a^	171.5 ± 10.4^a^	175.5 ± 3.5^a^	167.1 ± 16.1^a^	169.5 ± 9.5^a^
Karmen/mL						
ALT	133.1 ± 5.2^a^	143.9 ± 8.4^a^	146.6 ± 4.6^a^	141.9 ± 3.8^a^	138.8 ± 6.6^a^	146.3 ± 7.1^a^
Karmen/mL						

Data express the mean ± S.E. The letters in the columns indicate statistical differences by Duncan’s multiple range test (*p* < 0.05). Control: Normal rat supplied with tap water, BPH: Castrated rat supplied with tap water, AG 100: Castrated rat supplied with AG 100 mg/kg of BW daily, AG 250: Castrated rat supplied with AG 250 mg/kg of BW daily, AG 500: Castrated rat supplied with AG 500 mg/kg of BW daily. Data are represented as mean±SEM. a, P<0.05 vs. control, b, P<0.01 vs. control, c, P<0.001 vs. control

#### BPH related hormones (testosterone and DHT) in the blood

**Fig. 1:**
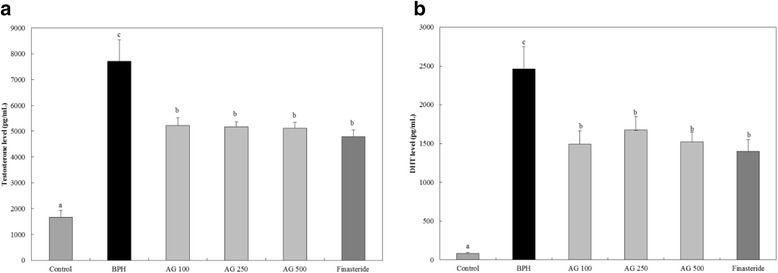
Effects of AG on DHT and testosterone levels in serum (**a**) Testosterone level (**b**) DHT level. Control: water, BPH: 3 mg.kg testosterone propionate, AG 100, AG 250, AG 500: 3 mg/kg testosterone propionate + AG 100 mg/kg, AG 250 mg/kg, AG 500 mg/kg, respectively, Finasteride: 3 mg/kg testosterone propionate + finasteride 10 mg/kg. Data represented as mean ± SD (*n* = 7). Significant difference at *p* < 0.05 compared with the Control group and BPH induced group, respectively

AG and finasteride treated groups showed a significantly lower testosterone and DHT concentration compared to the BPH induced group (Fig. 1).

### BPH-related gene expression

**Fig. 2:**
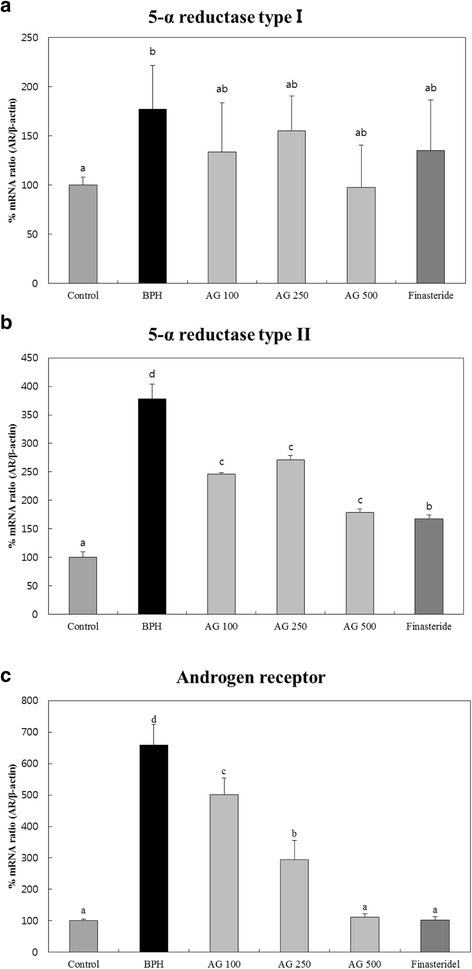
Effects of AG on expression of BPH-related gene’s mRNA in the prostate (**a**) 5-α reductase type I (**b**) 5-α reductase type II (**c**) Androgen receptor. Control: water, BPH: 3 mg/kg testosterone propionate, AG 100, AG 250, AG 500: 3 mg/kg testosterone propionate + AG 100 mg/kg, AG 250 mg/kg, AG 500 mg/kg, respectively, Finasteride: 3 mg.kg testosterone propionate + finasteride 10 mg/kg. Data represented as mean ± SD (*n* = 7). Significant difference at *p* < 0.05 compared with the Control group and BPH induced group, respectively

5-α reductase type I gene expression of AG and finasteride treated groups was significantly lower than the BPH induced group (Fig. 2a). Also, 5-α reductase type II gene expression of AG and finasteride treated groups was significantly reduced compared to BPH induced group (Fig. 2b).

In the AG-treated groups, the AR gene expression was decreased in a concentration-dependent manner. AR gene expression of AG 500 mg/kg/day treated group is as effective as finasteride treatment (Fig. 2c).

### Inflammation-related gene expression

**Fig. 3:**
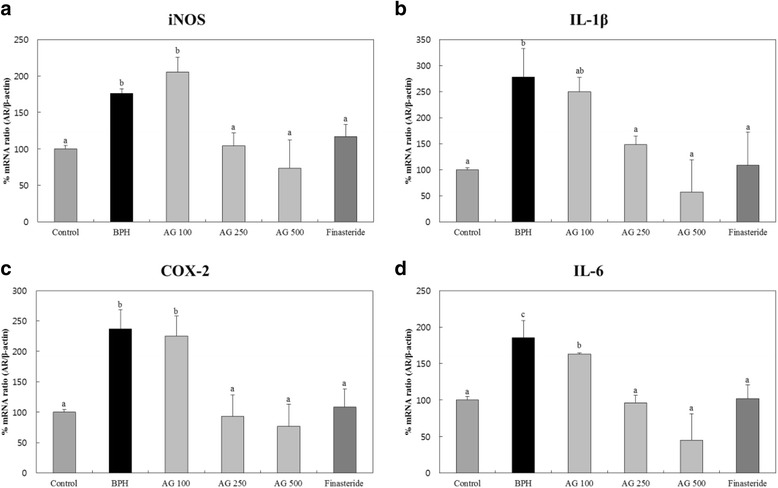
Effects of AG on expression of Inflammation-related gene’s mRNA in the prostate (**a**) iNOS (**b**) IL-1β (**c**) COX-2 (d) IL-6. Control: water, BPH: 3 mg/kg testosterone propionate, AG 100, AG 250, AG 500: 3 mg/kg testosterone propionate + AG 100 mg/kg, AG 250 mg/kg, AG 500 mg/kg, respectively, Finasteride: 3 mg.kg testosterone propionate + finasteride 10 mg/kg. Data represented as mean ± SD (*n* = 7). Significant difference at *p* < 0.05 compared with the Control group and BPH induced group, respectively

The iNOS gene expression level of AG 500 mg/kg/day treated group was lower than control group. Also, the expression was decreased in a concentration-dependent manner (Fig. 3a).

Likewise, the gene expression of IL-1β, COX-2 and IL-6 of AG 500 mg/kg/day treated group was lower than control group and decreased in a concentration-dependent manner (Fig. 3b–d). Finasteride treated group showed similar expression level of inflammation-related gene compared to control group.

### Western blot analysis of the prostate

#### BPH-related protein expression rate

**Fig. 4:**
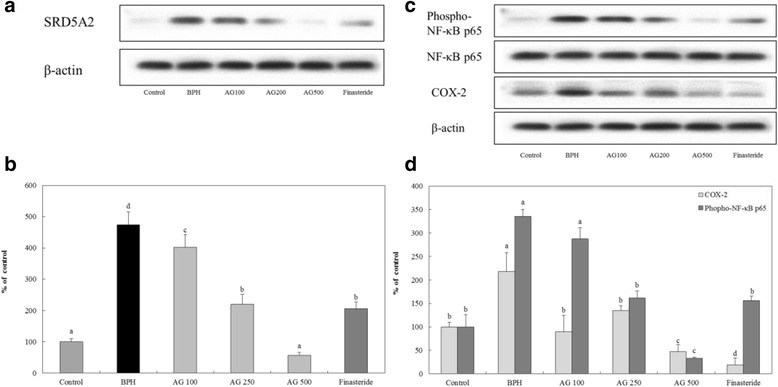
Effects of AG on expression of 5-α reductase II, COX-2 and NF-κB p65 phosphorylated protein in the prostate. **a** Expression of SRD5A2 (steroid 5-α reductase II) (**b**) The relative density of SRD5A2 was analyzed by densitometry. **c** Expression of COX-2, phosphorylated NF-κB p65. **d** The relative density of COX-2 and phosphorylated NF-κB p65. Control: water, BPH: 3 mg.kg testosterone propionate, AG 100, AG 250, AG 500: 3 mg/kg testosterone propionate + AG 100 mg/kg, AG 250 mg/kg, AG 500 mg/kg, respectively, Finasteride: 3 mg/kg testosterone propionate + finasteride 10 mg/kg. Data represented as mean ± SD (*n* = 7). Significant difference at *p* < 0.05 compared with the Control group and BPH induced group, respectively

The protein expression level of 5-α reductase II in the control group was significantly decreased from that of the BPH induced group. All of the AG-treated groups had a significantly decreased expression level that was dependent on the dose. Compared to finasteride treated group, the AG 250 and 500 mg/kg/day treated groups showed more reduced expression level of 5-α reductase II (Fig. 4a and b).

#### Inflammation-related protein expression rate

The BPH induced group had a significantly increased level of COX-2 protein expression and NF-κB phosphorylation compared to the control group. Otherwise, the level of expression and phosphorylation in AG-treated groups was gradually decreased compared to BPH induced group. Furthermore, AG 500 mg/kg/day treated group’s level of each protein showed less than finasteride treated group (Fig. 4c and d).

### Anti-oxidant indicators in the prostate

In BPH induced group, anti-oxidative ability was significantly decreased than control group. However, the ability of AG treated group was increased in a concentration-dependent manner compared to BPH induced group. AG 500 mg/kg/day treatment has as effective as finasteride (Table 4).

**Table 4:** Anti-oxidant indication in prostate organs

Groups	CAT	SOD	GST	GR	GPx	GSH
	(U/mg protein)	(U/mg protein)	(mU/mg protein)	(U/mg protein)	(U/mg protein)	(μmoles/mg protein)
Control	372.1 ± 7.4^a^	11.0 ± 1.1^a^	184.3 ± 9.6^a^	28.8 ± 1.1^a^	3.0 ± 0.2^a^	72.8 ± 3.1^a^
BPH	28.0 ± 3.7^d^	1.2 ± 0.2^c^	7.6 ± 3.0^d^	9.8 ± 0.3^c^	1.5 ± 0.1^c^	33.4 ± 3.2^c^
Finasteride	399.9 ± 38.5^b^	11.1 ± 0.2^a^	141.8 ± 11.7^ab^	24.6 ± 0.4^a^	2.7 ± 0.1^a^	67.6 ± 3.2^ab^
AG 100	35.4 ± 10.0^d^	6.0 ± 0.5^b^	74.0 ± 10.1^c^	19.9 ± 1.8^b^	1.9 ± 0.3^b^	34.7 ± 2.1^c^
AG 250	170.5 ± 10.9^c^	7.9 ± 0.8^b^	71.0 ± 23.8^c^	25.8 ± 2.3^a^	2.7 ± 0.1^a^	37.4 ± 0.9^c^
AG 500	232.0 ± 11.2^b^	11.2 ± 0.2^a^	172.2 ± 22.1^a^	26.5 ± 0.6^a^	2.7 ± 0.1^a^	62.0 ± 2.6^b^

Data express the mean ± S.E. The letters in the columns indicate statistical differences by Duncan’s multiple range test (*p* < 0.05). Control: Normal rat supplied with tap water, BPH: Castrated rat supplied with tap water, AG 100: Castrated rat supplied with AG 100 mg/kg of BW daily, AG 250: Castrated rat supplied with AG 250 mg/kg of BW daily, AG 500: Castrated rat supplied with AG 500 mg/kg of BW daily. CAT: Catalase, SOD: Superoxide dismutase, GST: Glutathione S-transferase, GR: Glutathione reductase, GPx: Glutathione peroxidase, GSH: Glutathione

### H&E staining

To verify the effectiveness of AG in mitigating prostate hypertrophy, H&E staining was performed and histological variation was observed.

**Fig. 5:**
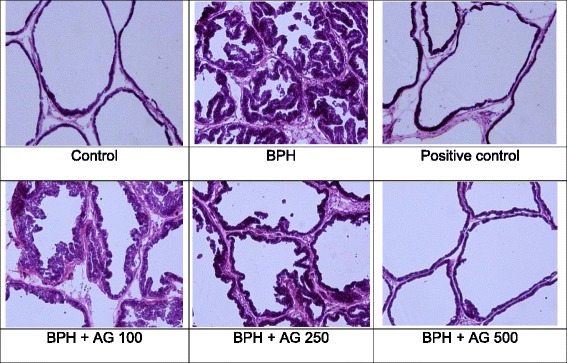
Hematoxylin and eosin (H&E) staining of prostate tissue. The prostate tissue sections were examined under an optical microscope at × 100 magnification (Olympus, Tokyo, Japan). Control: corn oil, BPH: TP + corn oil, Finasteride: TP + finasteride (10 mg/kg of bw daily), BPH + AG 100, 250, 500 mg: TP + water extract of *Acorus gramineus* Soland (100, 250 and 500 mg/kg of bw daily)

As shown in Fig. 5, the epithelial cells in the prostates of the BPH induced group became hyperplastic and the lacuna became narrowed. The level of epithelial cell hyperplasia in the prostates of the finasteride-treated group decreased compared to that of the BPH induced group and lacuna narrowing improved to the same extent as that of the control group. As the concentration increased in the AG groups, the level of epithelial cell hyperplasia decreased. In addition, in the group treated with the high concentration of AG lacuna narrowing was abated to the same extent as that of the control group and it had a similar shape as that of the control group.

## Discussion

The prostate is one of the male reproductive organs that produce part of the semen. Prostatic tissue is composed of four parts: epilepsy, line, epithelium and lumen. Histologically, BPH is a disease caused by the proliferation of stroma composed of the superficial follicular, connective tissue and smooth muscle. The BPH are caused by the compression of the prostate urethra due to the mechanical factors caused by the increase in prostate size and dynamic factors caused by the tension of the smooth muscle in the prostate [4, 22, 23].

The representative BPH therapeutic agent, finasteride, reduces the size of the prostate gland by inhibiting the production of DHT that is involved in the proliferation of the prostate gland [24]. However, even after long term administration, the size reduction didn’t exceed 30% in most cases. Moreover, even if it persisted longer, no further reduction could be expected. Therefore, the need for a safe alternative therapy has been raised [25].

*Acorus gramineus* is known to be effective for memory improvement, cataracts, hair and scalp health. It is perennial plant which is containing 0.5 ~ 0.9% of essential oil in roots. *A. gramineus* is enriched β-asarone, α-asarone, GABA, caryophyllen, phenolic compound [8, 9]. A recent our laboratory study confirmed the efficacy of BPH in vitro and will soon be published in the paper. *A. gramineus* is known to be toxic to mutation and carcinogenesis. Most of these are essential oils and are typically β-asarone. We used hot-water extract of *A. gramineus* root to remove almost toxic volatile substances. Because the toxic substance was removed by hot water extract and the efficacy of AG was confirmed in previous experiment, we studied *A. gramineus* for BPH.

In the present study, the prostate weight and body weight were significantly increased in the BPH-induced group compared to the control group. This shows that the BPH is properly formed in this experimental model. As a result of observation of body weight change, statistically significant decrease was observed in BPH-induced group, AG treated group and finasteride treated group compared to control group. In the case of AG and finasteride, the experimental group was stimulated by oral administration and weight loss was thought to be caused by such stimulation [16]. Despite weight loss, AG is thought to have antioxidant effect through various antioxidant indicator results (Table 4). As a result, the prostate weight and prostate/body weight ratio were significantly decreased in the AG-treated group, suggesting that AG is effective for enlargement of the prostate.

BPH is related to the male hormones, testosterone and DHT. Testosterone is converted to activated DHP by 5-α reductase in the prostate and the activated DHT binds to androgen receptor on prostate cell to induces BPH [19]. Our results show that AG reduces the testosterone and DHT level in blood (Fig. 1). mRNA level of BPH related genes also decreased (Fig. 2) [26]. BPH is concomitant with inflammation and NF-κB inflammatory cytokines are activated. When BPH induces inflammation, oxygen free radicals are created and cause oxidative stress [27]. Therefore, the biomarkers, prostate weight ratio, hormonal changes, 5-α reductase type II androgen receptor and anti-oxidant activation factors, were chosen to evaluate the effectiveness of AG for BPH. We confirmd the effect of AG in these markers and elucidated that AG is an effective material in BPH and reactive oxygen species (ROS).

## Conclusion

AG is effective in inhibiting the BPH compared to the finasteride treatment group. Thus, it can be used as an field of the BPH treatment by supplementing the problems of the existing treatment methods. Based on the results of this study, additional clinical studies are needed for patients with BPH.

## Abbreviations

AG: *Acorus gramineus* root hot water extract
ALT: Alanine aminotransferase
ANOVA: Analysis of variance
AR: Androgen receptor
AST: Aspartate aminotransferase
BPH: Benign prostatic hypertrophy
COX-2: Cyclooxygenase-2
DAD: Diode array detector
DHT: Dihydrotestosterone
FER: Food efficiency ratio
GABA: γ-aminobutyric acid
GPx: Glutathione peroxidase
GR: Glutathione reductase
GSH: Glutathione
GST: Glutathione S-transferase
H&E: Hematoxylin and eosin
HPLC: High performance liquid chromatography
IL: Interleukin
iNOS: Nitric oxide synthase
Na_2_HPO_4_: Sodium phosphate dibasic anhydrous
NF-κB: Nuclear factor kappa-light-chain-enhancer of activated B cells
RT-PCR: Real-time polymerase chain reaction
SDS-PAGE: Sodium dodecyl sulfate-polyacrylamide gel electrophoresis
SEM: Stander error of the mean
SOD: Superoxide dismutase
TP: Testosterone propionate
